# Protective efficacy of anti-neuraminidase monoclonal antibodies against H7N9 influenza virus infection

**DOI:** 10.1080/22221751.2019.1708214

**Published:** 2020-01-02

**Authors:** Fei-Fei Xiong, Xue-Ying Liu, Fei-Xia Gao, Jian Luo, Peng Duan, Wen-Song Tan, Ze Chen

**Affiliations:** a Shanghai Institute of Biological Products, Shanghai, People’s Republic of China; b East China University of Science and Technology, Shanghai, People’s Republic of China

**Keywords:** H7N9 influenza virus, neuraminidase, monoclonal antibodies, protection, NA epitope

## Abstract

The H7N9 influenza virus has been circulating in China for more than six years. The neuraminidase (NA) has gained great concern for the development of antiviral drugs, therapeutic antibodies, and new vaccines. In this study, we screened seven mouse monoclonal antibodies (mAbs) and compared their protective effects against H7N9 influenza virus. The epitope mapping from escape mutants showed that all the seven mAbs could bind to the head region of the N9 NA close to the enzyme activity sites, and four key sites of N9 NA were reported for the first time. The mAbs D3 and 7H2 could simultaneously inhibit the cleavage of the sialic acid of fetuin protein with large molecular weight and NA-XTD with small molecule weight in the NA inhibition experiment, prevent the formation of virus plaque at a low concentration, and effectively protect the mice from the challenge of the lethal dose of H7N9 virus.

## Introduction

On 29 March 2013, the Chinese Center for Disease Control and Prevention confirmed the first human case of the H7N9 avian influenza virus infection [1]. Until now, the H7N9 virus has been circulating in China for more than six years, with the case fatality rate of nearly 30%, characterized by severe lung disease and acute respiratory distress syndrome (ARD) [2] and with the emergence of drug-resistant and highly pathogenic strains. The H7N9 virus has a high replication capacity in the respiratory tract cells of mammals and humans. Since most human infections were linked to direct contact with live poultry, the local governments of China have implemented control measures to reduce the prevalence of H7N9 virus in poultry markets, such as conducting environmental sampling and laboratory tests of poultry markets [3]. Every spring, the live poultry markets in some cities also were closed [4]. The measures have been effective in reducing the risk of H7N9 virus transmission.

Most influenza vaccines currently in use are the inactivated influenza virus split vaccines containing a mixture of influenza virus proteins, including haemagglutinin (HA) and neuraminidase (NA) of the viral surface glycoprotein which are responsible for virus attachment and release from the host cells [5]. NA is the second major glycoprotein on the surface of influenza viruses in the form of a tetramer. Each subunit consists of four domains, a short and highly conserved N-terminal cytoplasmic sequence, a hydrophobic transmembrane domain, a stem region, and a globular “head” domain carrying an enzyme activity site. A total of nine NA subtypes of the influenza virus have been discovered, which can be classified into group 1 (N1, N4, N5, N8) and group 2 (N2, N3, N6, N7, N9) NAs [6]. Past studies have demonstrated that NA catalyses the terminal sialic acid residues from the newly formed virions and from the host cell receptors to assist in the mobility of virus particles through the respiratory tract mucus and in the elution of virion progeny from the infected cell [7,8].

Mouse monoclonal antibodies (mAbs) have been increasingly used to prevent or treat viral infections. For influenza virus, the anti-influenza virus mAbs are mostly targeting various epitopes of HA or matrix protein 2 (M2). Besides, anti-NA mAbs have also been shown to block virus growth and protect against challenge with a lethal virus in mouse models [9,10], including some N9 NA mAbs, such as 3c10-3 [11,12], 1E8, 2F6, 10F4, 11B2 [13]. It had been reported that there are roughly 30 essential amino acids in the protective epitopes of NA in the subtypes N2, N8, and N9 [14]. However, one possible concern regarding mAbs for the therapeutic purpose was that mAbs might drive the evolution of viral escape mutations, resulting in resistance to mAb or vaccine-induced immunity. Our previous series of studies also confirmed that the NA specific immune response could resist the attack of the influenza virus. The mice immunized with NA-DNA were induced to produce high levels of specific antibody responses and provided protection against the challenge from the lethal doses of influenza virus [15–25].

In this paper, we prepared seven NA mAbs (5F2, 2H10, 7H2, D3, F6, 7D8, B7C2) of the influenza virus A (H7N9). We detected their NI inhibition and plaque inhibition abilities, identified their epitopes, and tested their preventive and therapeutic effects in a mouse model. The correlation between the *in vitro* and *in vivo* activity of the NA mAbs was also compared, as well as the four key epitopes of N9 NA were reported for the first time.

## Materials and methods

### Cells, viruses, plasmid DNA

Madin-Darby canine kidney (MDCK) cells and HEK 293 T cells were maintained in our lab. SP2/0 mouse myeloma cells were purchased from ATCC (Sp2/0-Ag14; ATCC CRL-1581). All cells were grown in complete Dulbecco’s modified Eagle medium (DMEM; Life Technologies, US) supplemented with 10% foetal bovine serum (FBS; Gibco, US).

Influenza viruses used in this study were mouse-adapted H7N9 (A/Shanghai/2/2013, SH/2/13), H9N2 (A/chicken/Hunan/2/2008, HN/2/08) and H1N1 (A/Puerto Rico/8/1934, PR8) viruses, which were grown in 8–10-day-old embryonated chicken eggs, and titres were determined on MDCK cells in the presence of TPCK (tolylsulfonyl phenylalanyl chloromethyl ketone)-treated trypsin (Sigma, US).

pCAGGSP7/NA was constructed by cloning the NA gene from the influenza virus strain A/Shanghai/2/2013 (H7N9) into expression vector pCAGGSP7, as described in our previous studies [15–20]. The plasmid was propagated in *Escherichia coli* XL1-blue bacteria and purified using Qiagen Purification Kits (Qiagen, US).

### Generation and screening of mAbs

*In vivo* electroporation was carried out according to the method described previously [15–20,26]. Female BALB/c mice (aged 4 weeks) were immunized three times, at an interval of 2 weeks, by injection with 50 μg NA DNA plasmid using an electric-pulse generator (Electro Square Porator T830 M; BTX, San Diego, CA, USA). On day 3 before the fusion, one mouse was boosted with 50 μg NA DNA plasmid by the tail vein injection. Splenocytes from immunized mice were fused with Sp2/0 cells. Hybridomas were screened with enzyme-linked immunosorbent assays (ELISAs) using the harvested virus suspension of SH/2/13 (H7N9)-coated plates. Positive clones were subcloned twice by limiting dilution. Each hybridoma was grown in serum-free medium, and representative mAbs were purified using protein G columns (GE Healthcare, Uppsala, Sweden).

### Enzyme-linked immunosorbent assays (ELISA)

Ninety-six-well plates were coated overnight with 5 μg/ml (50 μl/well) of H7N9 virus at 4°C. The coating buffer was discarded, and the plates were blocked with 2% milk in phosphate buffer saline (PBS; 100 μl/well) for 1 h at room temperature. In the case of hybridoma screening, 100 μl of undiluted supernatant from each hybridoma clone was added directly to wells. In the case of detecting the ability of mAbs to bind to the H7N9 virus, mAbs were serially diluted at a starting concentration of 1 μg/ml. The plates were then incubated for 1 h at room temperature. After three washes with PBS (100 μl/well for each wash), the plates were incubated for another hour at room temperature with horseradishperoxidase (HRP)-labeled anti-mouse antibody (1:3000; KPL, US; 100 μl/well) and the signal was developed using tetramethylbenzidine (TMB) as the substrate. The reaction was stopped with sulphuric acid, and the optical density at 450 nm (OD_450_) was read.

### NA enzyme-linked lectin assay (ELLA)

To determine the optimal concentrations of viruses for the NI assays, the data of NA activities of virus were analysed by GraphPad Prism 5.0 and fit to a nonlinear curve as previously described [27]. The optimal concentrations of viruses EC_50_ (50% effective concentration) was obtained in OD values of around 1.0, half the maximal OD value in the ELISAs. The inhibition of NA enzyme activity by mAbs was measured with an enzyme-linked lectin assay (ELLA) in 96-well plate as described previously [28]. Serial dilutions of mAbs were mixed with the H7N9 SH/2/13 virus diluted with 1% bovine serum albumin (BSA) in PBS containing Tween 20 (PBST). The mixture was transferred to 96-well plates coated with fetuin (Sigma-Aldrich, US) and incubated overnight at 37°C. Plates were washed with PBST, followed by the addition of peanut agglutinin conjugated to HRP (Sigma-Aldrich, US). Plates were incubated at room temperature for 2 h in the dark and then washed with PBST before the addition of TMB substrate. The reaction was stopped, and OD_450_ values were read. The values were divided by the average value for virus-only control wells and then multiplied by a factor of 100 to obtain the NA activity. Percent inhibition was calculated by subtracting the NA activity from 100. The median inhibition concentration (IC_50_) was calculated as the inverse dilution of antibody that resulted in 50% inhibition of NA activity.

### NA-XTD assay

The NA-XTD assay was performed according to NA-XTD™ Influenza Neuraminidase Assay Kit manufacturer’s instructions (Applied Biosystems, life, US). Briefly, 25 μl test mAbs in serial two-fold dilutions in NA-XTD assay buffer were mixed with 25 μl of 4 × EC_50_ of H7N9 virus and incubated at 37°C for 20 min. After adding 25 μl NA-XTD substrate, the plates were incubated at room temperature for 30 min. The reaction was stopped by adding 60 μl of NA-XTD Accelerator. The chemiluminescent was determined by using the SpectraMax i3× reader (MD, US). Data points were analysed using Graphad Prism 5.0 software and the 50% inhibition concentration (IC_50_) was defined as concentration at which 50% of the NA activity was inhibited compared to the negative control.

### Plaque reduction assay

MDCK cells in six-well plates were inoculated with ∼30–40 plaque-formation units (PFUs) of H7N9 virus. After incubated at 37°C for 1 h, cells then were overlaid with agar supplemented with various concentrations of mAbs. Cells inoculated with virus and overlaid with agar without mAbs were set up as controls. Cells were stained with crystal violet solution to visualize plaques on 72 h after virus inoculation.

### Escape mutants

N9 mAb escape variants were selected as previously reported [29]. Briefly, 4 mg of each mAb was mixed with 10^6^ PFU of H7N9 SH/2/13 virus. After incubation at room temperature for 30 min, the mixture was inoculated into 8–10-day-old embryonated chicken eggs (5 eggs/mAb). After 3 days of incubation, allantoic fluid was collected and confirmed by a haemagglutination assay. If positive, the potential escape variants were inoculated onto MDCK cells for plaque assay in the presence of the selecting mAb. Plaques were picked and propagated in eggs. The NA gene of each variant was amplified by RT–PCR and PCR products were sequenced. Sequences were analysed to identify nucleotide and amino acid changes. The structure was generated with Chimera software (RBVI).

### Immunofluorescence assay (IFA)

293 T cells growing in 24 well plates were transfected with NA plasmids expressing NA of H7N9 SH/2/13 virus. MDCK cells growing in 24 well plates were infected with H7N9 SH/2/13 virus. 293 T and MDCK cells were incubation at 37°C in 5% CO_2_ for 48 h. Cells were fixed with 3.7% paraformaldehyde, permeabilized with 0.1% Triton X-100 (Sigma-Aldrich, USA), and then incubated with each mAb, followed by incubation with Alexa Fluor^®^488 – conjugated anti-mouse antibody (Jackson ImmunoResearch, US). The mAb gH19 (IgG2a) against varicella-zoster virus was used as a negative control. Nuclei were stained with DAPI (Beyotime Biotechnology, China). Cells were then observed under immunofluorescence microscope (Olympus, Japan).

### Flow cytometry analysis (FCM)

MDCK cells infected with H7N9 virus in suspension were incubated with anti-N9 NA mAbs or a negative control mAb for 45 min on ice. After washing 3 times with PBS, Alexa Fluor™ 488-conjugated anti-mouse antibody (Jackson ImmunoResearch, US) was added and incubated for 30 min on ice. After washing, the number of infected cells was determined by flow cytometric analysis on an Attune NxT acoustic focusing cytometer (Life Technologies, US). The mAb gH19 (IgG2a) against varicella-zoster virus was used as a negative control.

### Evaluation of the prophylactic and therapeutic efficacy in mice

In prophylactic experiment, groups of five female BALB/c mice aged 6 weeks received a 50, 30, or 10 mg/kg dose of each purified mouse monoclonal antibody intraperitoneally (i.p.). At 24 h after treatment, the mice were anaesthetized using a ketamine-xylazine mixture and infected intranasally (i.n.) with the 5 × LD_50_ (50% lethal dose) of H7N9 virus diluted in PBS. In a therapeutic setting, mice also received the 50, 30, 10 mg/kg dose of each antibody intraperitoneally 24 h after infection. The mAb gH19 (IgG2a) against varicella-zoster virus was used as a negative control. Mice that received a 50 mg/kg antibody dose prophylactically were sacrificed either on day 3, 5, and 7 postinfection for lung titre analysis (*n* = 3). At these time points, the lungs were harvested and homogenized using a BeadBlaster 24 (Benchmark, US) homogenizer, and viral lung titres were measured by plaquing lung homogenates on MDCK cells. In all other groups, mice were monitored daily for survival and weight loss until day 20 postinfection. Mice that lost more than 35% of their initial body weights were euthanized.

### Ethics statement

Specific-pathogen-free female BALB/c mice (4–5 weeks old) were purchased from Shanghai Laboratory Animal Center, China. All mice were bred in biosafety level 3 (BSL3) or the Animal Resource Center at Shanghai Institute of Biological Products (SIBP), and maintained in specific-pathogen-free conditions. All experiments involving animals were approved by Animal Care Committee of SIBP (Protocol Number 14–5639), in accordance with the animal ethics guidelines of the Chinese National Health and Medical Research Council (NHMRC).

## Results

### Generation of NA mAbs against H7N9 virus

**Figure 1.**
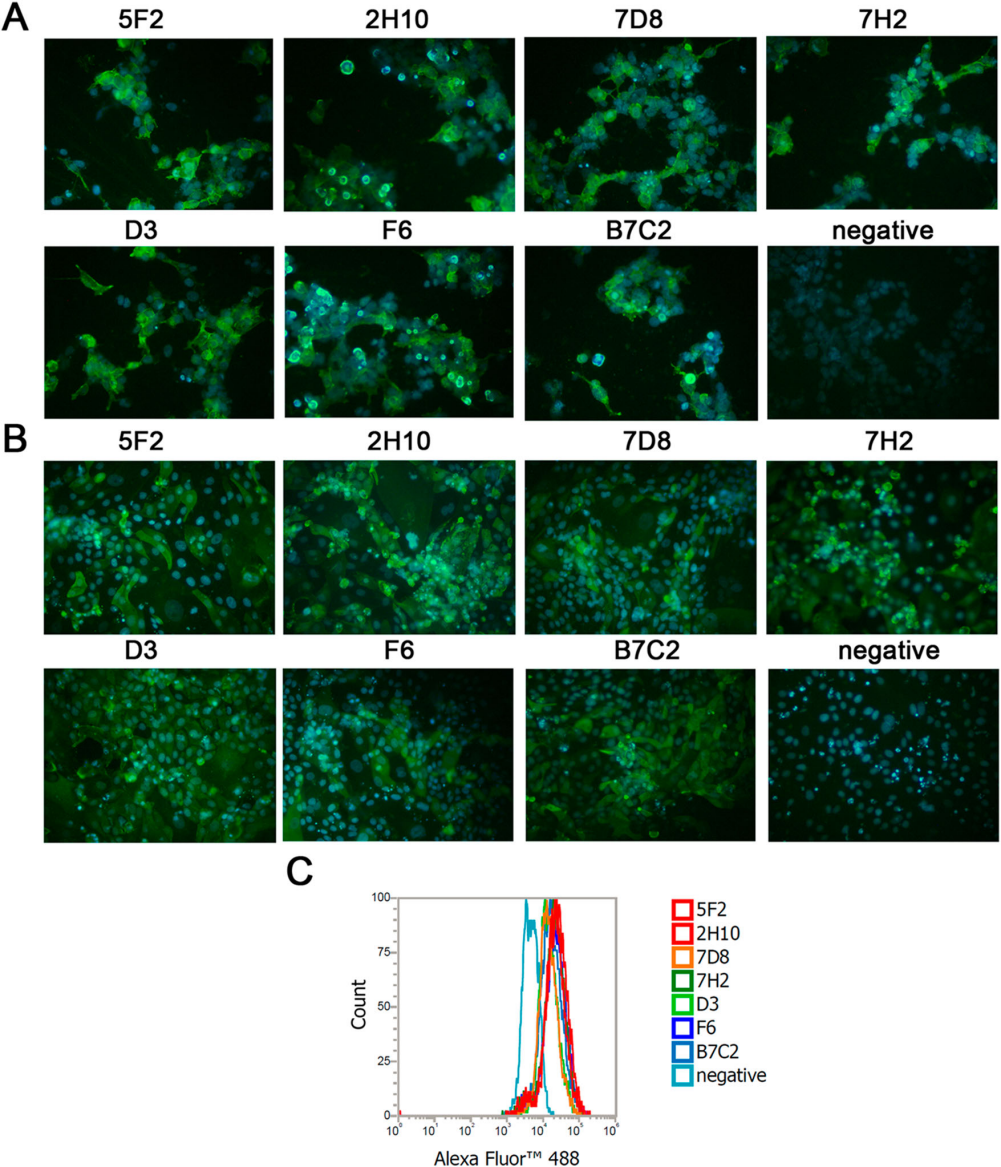
Characterization of N9 mAbs in IFAs and FCM analysis. (A) 293 T cells were transfected with NA plasmids expressing NA of H7N9 SH/2/13 virus. (B) MDCK cells were infected with H7N9 SH/2/13 virus. (C) The representative graph of MDCK cells infected with SH/2/2013 virus. Bound antibodies were detected by Alexa Fluor™ 488-conjugated anti-mouse antibody. An isotype-matched IgG (IgG2a) was used as a negative control. Nuclei were stained with DAPI (blue).

We selected seven mAbs against the NA of H7N9 SH/2/13 virus generated through a fusion of Sp2/0 myeloma cells and splenocytes from mice immunized with NA plasmids. All mAbs were IgG2a isotype. To confirm the binding specificities, mAbs 5F2, 2H10, 7D8, 7H2, D3, F6, and B7C2 were tested by IFA with 293 T cells expressing NA of H7N9 SH/2/13 virus (Figure 1(A)), and MDCK cells infected with H7N9 SH/2/13 virus (Figure 1(B)). FCM analysis was also used to distinguish MDCK cells infected or uninfected with H7N9. As depicted in Figure 1(C), following treated with seven mAbs stained with Alexa Fluor™ 488, mAbs could increase the fluorescence intensity, which meant all seven mAbs could all bind to H7N9. In addition, all mAbs could react with H7N9 virus and NA of H7N9, but exhibited no reactivity with H9N2 (HN/2/08) and H1N1 (PR8) viruses (Table 1).

**Table 1.** Reactivity to the indicated viruses by ELISA and NA enzyme inhibition by ELLA and NA-XTD of N9 mAbs.

mAbs	ELISA (ng/ml)^a^			ELLA (μg/ml)^b^			NA-XTD (μg/ml)^b^		
	H7N9 (SH/2/13)	H9N2 (HN/2/08)	H1N1 (PR8)	H7N9 (SH/2/13)	H9N2 (HN/2/08)	H1N1 (PR8)	H7N9 (SH/2/13)	H9N2 (HN/2/08)	H1N1 (PR8)
5F2	0.98	>5000	>5000	21.2	>500	>500	96.7	>500	>500
2H10	1.95	>5000	>5000	42.4	>500	>500	>500	>500	>500
7D8	1.95	>5000	>5000	34.2	>500	>500	>500	>500	>500
7H2	0.49	>5000	>5000	4.74	>500	>500	35.1	>500	>500
D3	0.49	>5000	>5000	5.22	>500	>500	47.9	>500	>500
F6	0.98	>5000	>5000	19.1	>500	>500	215.2	>500	>500
B7C2	3.90	>5000	>5000	19.3	>500	>500	>500	>500	>500

^a^Data were shown as the minimum mAb concentration required to produce a 2-fold signal above negative control.

^b^Data were shown as the minimum concentration of antibody required to inhibit 50% of enzyme activity.

### N9 mAbs inhibit NA enzymatic activity

The enzymatic function of NA is to catalyse the hydrolysis of terminal sialic acid residues, assisting in the elution of virion progeny from the infected cell. To further determine the inhibitory capacity of the NA mAbs, neuraminidase inhibition activity was measured using ELLA and NA-XTD assays. For ELLA, we used fetuin (49 kD) as substrate, whose bulky glycans exist in the sialic acid substrate. When an antibody binds to NA, even outside the enzymatic site, it will block the substrate access to the enzymatic site because of steric hindrance. Instead, the NA-XTD substrate could easily enter the enzyme site of NA, unless the enzyme site was bound by the antibody. H7N9 SH/2/13 virus was used in both assays.

Shown in Table 1, mAbs 7H2 and D3 inhibited the reaction of both fetuin and NA-XTD substrate with NA of H7N9. 7H2 and D3 efficiently inhibited NA activity in ELLA, with IC_50_ < 10 μg/ml, while in NA-XTD assay, two mAbs required higher concentrations to inhibit NA, with IC_50_ between 30 and 50 μg/ml. MAbs 5F2 and F6 could also inhibit both substrates, but the concentrations needed in NA-XTD assay were relatively higher. Other mAbs affected the inhibition of NA activity by the size of the substrate. MAbs 2H10, 7D8, and B7C2 could inhibit the cleavage of fetuin with a large molecular weight, but failed to inhibit the ability of NA to cleave a small substrate (Table 1).

### N9 mAbs inhibit virus growth efficiently

**Figure 2.**
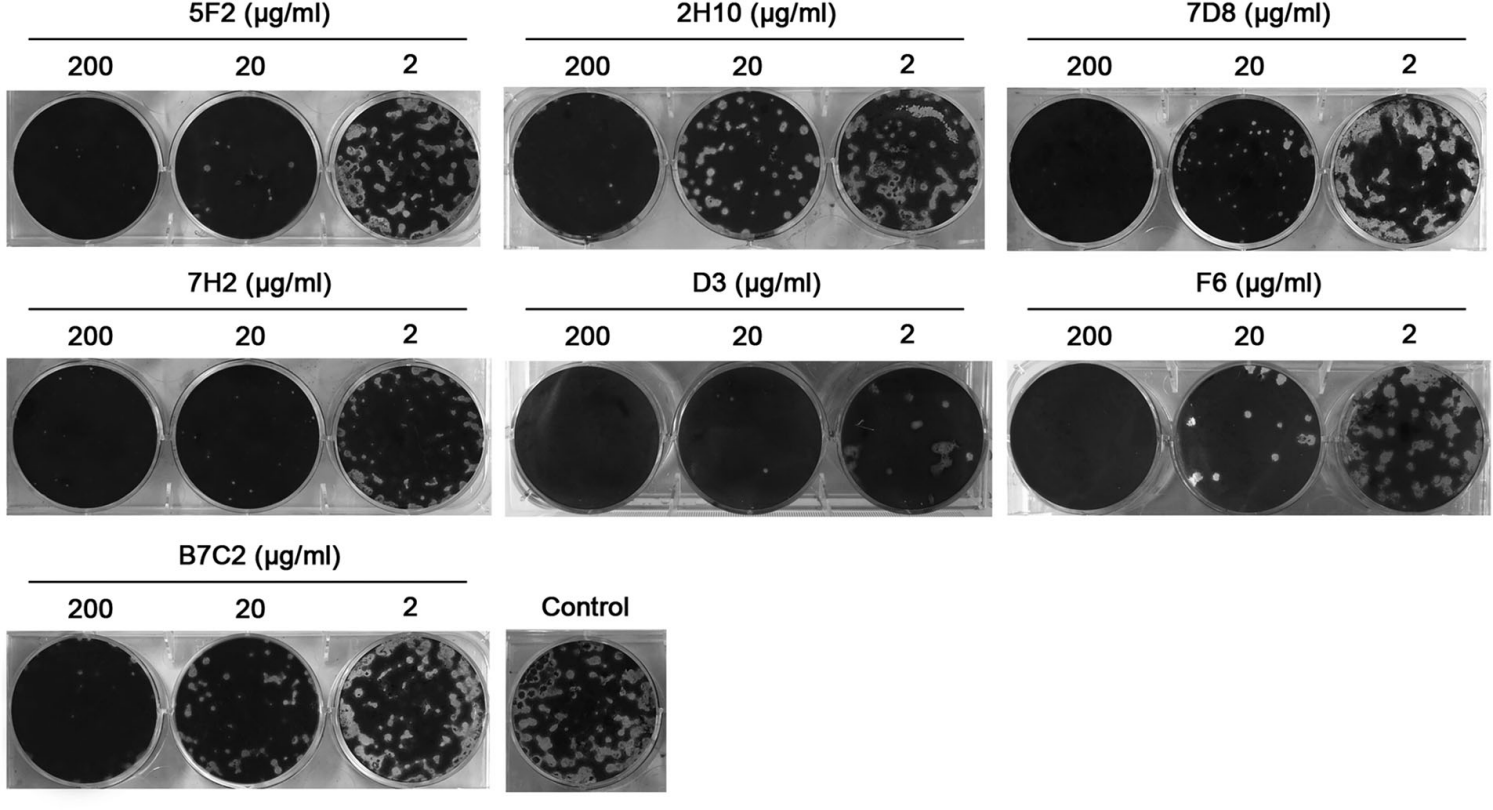
N9 mAbs inhibited H7N9 virus plaque formation. MDCK cells growing in six-well plates were infected with ∼30–40 plaque-formation units (PFUs) of H7N9 SH/2/13 virus, and then overlaid with agar that contained various concentrations of mAbs 5F2, 2H10, 7D8, 7H2, D3, F6, B7C2 (2, 20, 200 μg/ml) or without mAbs.

**Figure 3.**
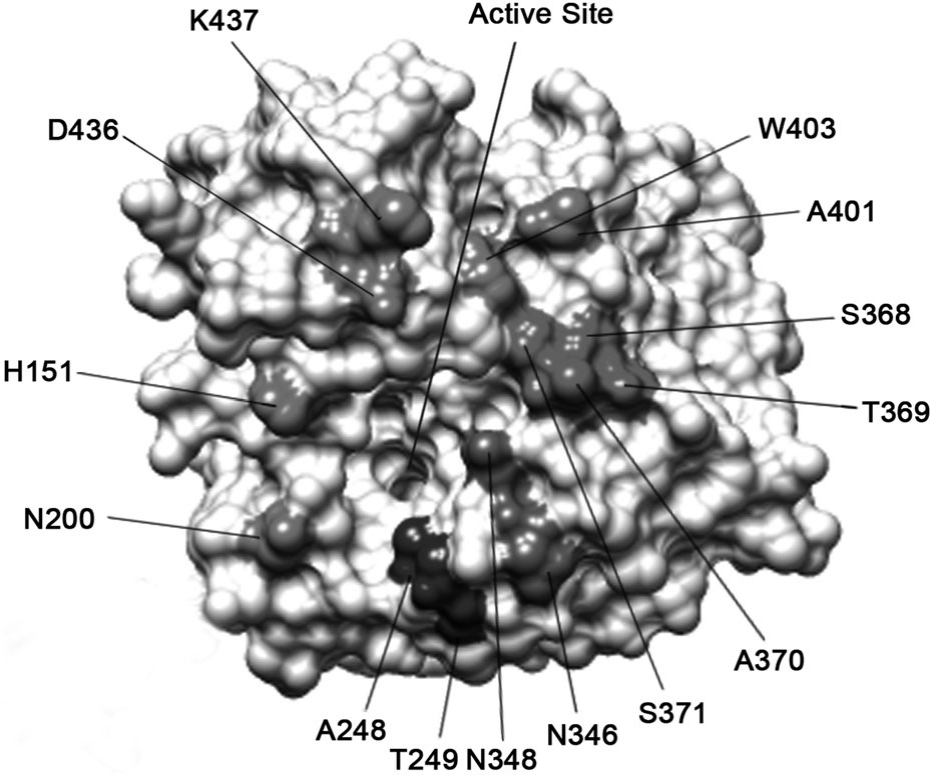
Schematic representation of the epitopes recognized by N9 mAbs on the H7N9 SH/2/13 NA molecule. Amino acid positions were designated in N9 numbering. Epitopes of NA mAbs were identified on the 3-dimensional structure of a NA monomer (PDB accession code 4MWL). The image was generated with Chimera software (RBVI).

We compared the effect of the mAbs on the growth of H7N9 SH/2/13 virus in MDCK cells by plaque assays. We observed that all mAbs significantly inhibited plaque formation or reduced H7N9 virus plaque size when the agar overlay was supplemented with either mAb (Figure 2). All mAbs could efficiently inhibit H7N9 virus plaque formation at concentrations of 200 μg/ml. When applied at 20 μg/ml, mAbs 5F2, 7H2, and D3 could inhibit virus to form pinpoint plaques, while there was a slight reduction in the size of plaques formed in the presence of other mAbs. Therefore, the plaque assays revealed that 5F2, 7H2, and D3 were more effective than other mAbs in inhibiting SH/2/13 virus proliferation, which was consistent with the ability of each mAb to inhibit NA activity (Figure 3).

### N9 mAbs bind near the enzyme active site of N9 NA

To identify the epitope residues critical for antibody binding, H7N9 SH/2/13 virus escape mutants were selected and plaque-purified in MDCK cells. H7N9 virus was propagated continuously in the presence of increasing concentrations of mAbs. Potential escape mutants were purified by plaques, and their NA genes were sequenced to identify putative amino acid substitutions that were resistant to mAbs. Each mAb selected two escape mutants (Table 2).

**Table 2.** Epitope mapping of N9 mAbs site-directed mutant NAs from H7N9 SH/2/13 by IFAs.

MAbs	Mutant site^a^	IFA^b^
5F2	A370T	−
	S371L	−
	WT	+
2H10	D436E	−
	K437R	−
	WT	+
7D8	P368S	−
	T369L	−
	WT	+
7H2	H151P	−
	N200S	−
	WT	+
D3	A248G	−
	T249N	−
	WT	+
F6	N346S	−
	N348S	−
	WT	+
B7C2	A401G	−
	W403K	−
	WT	+

^a^The amino acid mutant positions were in N9 numbering.

^b^IFAs were performed on 293 T cells transfected with mutant NA constructions. “+” or “−” indicated the presence or absence of fluorescence.

To evaluate the effects of these amino acid substitutions, each NA mutant was examined by IFA assays. Each selected amino acid was replaced by a corresponding variable amino acid identified in wild-type H7N9 virus strains, or a structurally similar amino acid. As shown in Table 2, mAbs either lost binding, or had a significant reduction in binding to the selected mutant viruses. These residues located on the top surface of NA and mostly were in close proximity to the enzyme active site. MAb D3 selected mutations at residues 248 and 249, which abolished or significantly reduced the binding of NA. This location was close to the active site. Once D3 bound to the NA, the NA activity was inhibited, which was consistent with the results of the NI assay. Similarly, the binding of mAbs 5F2, 2H10, 7D8, 7H2, F6, and B7C2 to NA was dramatically reduced by mutations screened by a corresponding antibody. The mutation sites selected by 5F2, 7H2, and F6 were also close to the active sites, with NI activity. Epitopes of other mAbs were a little far away from the active sites, with little NI activity.

To determine the evolutionary conservatism of the identified amino acid positions, we further aligned full-length sequences of NA from H7N9 virus strains available in the Influenza Research Database (http://www.fludb.org/brc/home.do?decorator=influenza) representing the major epidemic avian H7N9 genetic clades up to July 2018. Among 125 human H7N9 virus strains, all critical amino acid positions were relatively conserved, indicating that seven mAbs could recognize the majority of epidemic avian H7N9 viruses.

### N9 mAbs protected mice from lethal challenge with H7 influenza virus prophylactically or therapeutically

To determine whether potent *in vitr*o NA inhibitory efficacy would be predictive of prophylactic or therapeutic efficacy *in vivo*, female BALB/c mice were passively administrated (i.p.) 50, 30, and 10 mg/kg of mAbs 5F2, 2H10, 7D8, 7H2, D3, F6, and B7C2, respectively, before (prophylactically) or after (therapeutically) challenged i.n. with 5×LD_50_ of HPAI H7N9 SH/2/13 virus. We chose a dose of 5 × LD_50_ to ensure 100% mortality in the control group. The doses of mAbs used in this study were based on the preliminary studies with a less virulent virus.

**Figure 4.**
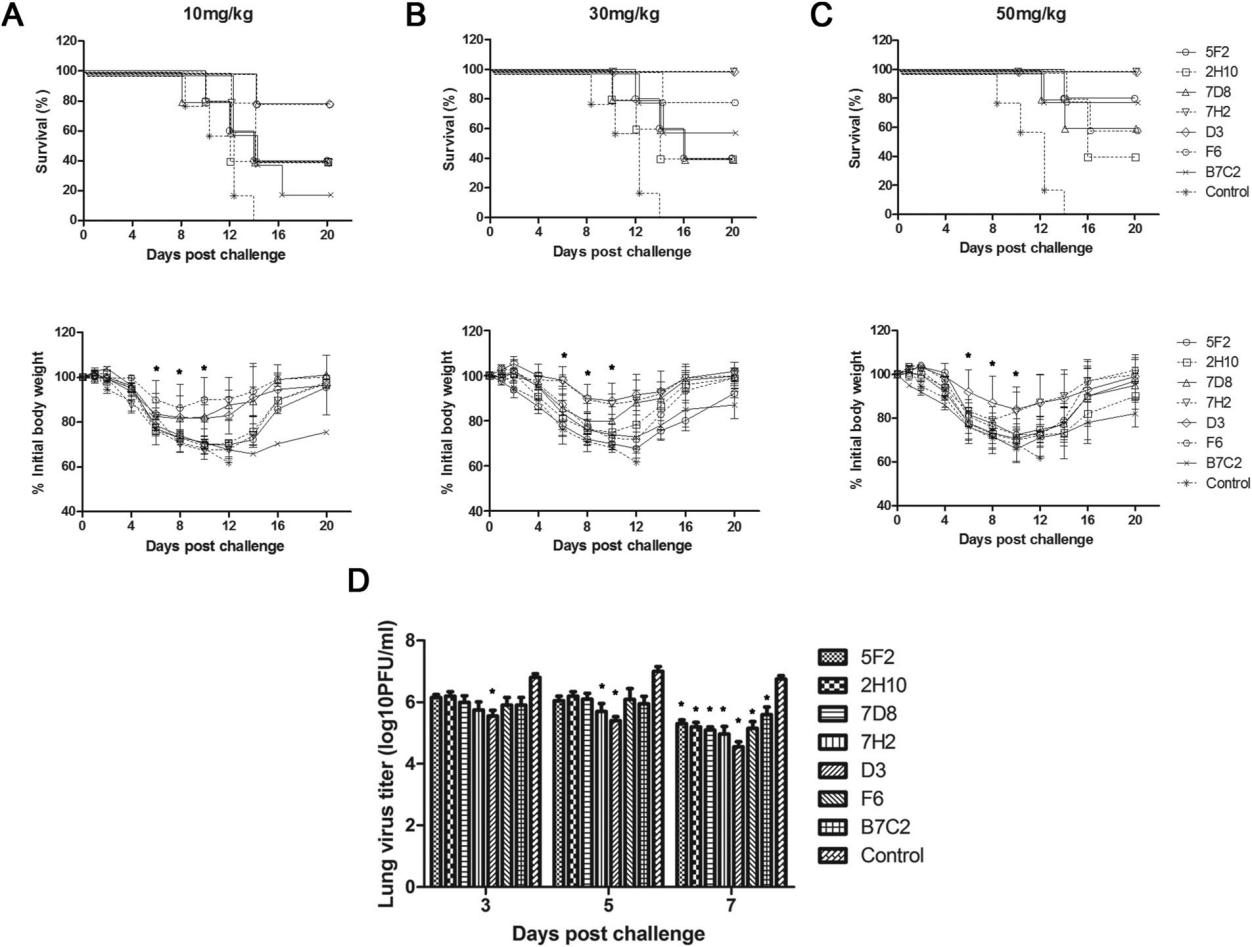
Prophylactic efficacy of N9 mAbs against a lethal H7N9 virus challenge in mice. BALB/c mice (*n* = 5 per group) were treated i.p. with NA mAbs at indicated doses, followed by challenging i.n. with 5 × LD_50_ of H7N9 SH/2/13 virus 24 h later. An isotype-matched and irrelevant mAb was used as a negative control. Survival (upper panel) and weight loss (lower panel) (*n* = 5 per group) were monitored for up to 20 days. (*, *p* < 0.05, 7D8 (10 mg/kg at 8, 10d), 7H2 (30 mg/kg at 6, 8, 10d; 50 mg/kg at 10d), D3 (10 mg/kg at 8, 10d; 30 mg/kg at 6, 8, 10d; 50 mg/kg at 6, 8, 10d), F6 (10 mg/kg at 6, 8, 10d) compared to the negative control by student *t*-test). (D) Lungs were collected on the 3, 5, and 7 d after infecting virus and viral titres were determined by titrating in MDCK cells. Titres were expressed as log10PFU/ml (*n* = 3); SD was shown with an error bar. Significant differences between titres measured in each group and control groups were shown as *, *p* < 0.05.

In the prophylaxis study, Figure 4 (upper panel) showed the survival rate and Figure 4 (lower panel) showed the time course of body weight changes for each group 20 days after challenge with H7N9 virus in different doses of mAbs. In the control group of mice, severe sickness became evident on day 3–5 after the challenge with the H7N9 virus, and all mice died. In contrast, all the groups of mice that were inoculated with different doses of mAbs got more or less protection post-challenge with virus. As shown in Figure 4, mAbs 5F2, 2H10, 7D8, 7H2, D3, and B7C2, especially 7H2, D3, conferred protection in a dose-dependent manner, with the greatest survival at the highest dose. 7H2 and D3 were the most effective, as either 30 or 50 mg/kg resulted in 100% protection with less weight loss. 5F2 provided 80% protection in animals at a dose of 50 mg/kg. 2H10 and 7D8 conferred 60% protection at 50 mg/kg dose. In the group of mice that were inoculated 50 mg/kg of with B7C2, only one mouse died, while the remaining 4 mice survived with poor health and gained little weight. F6 provided moderate protection (60–80%) at the doses of 10, 30, and 50 mg/kg (Figure 4). Taken together, 7H2 and D3 had the most effective prophylactic efficacy against H7N9 virus infection.

**Figure 5.**
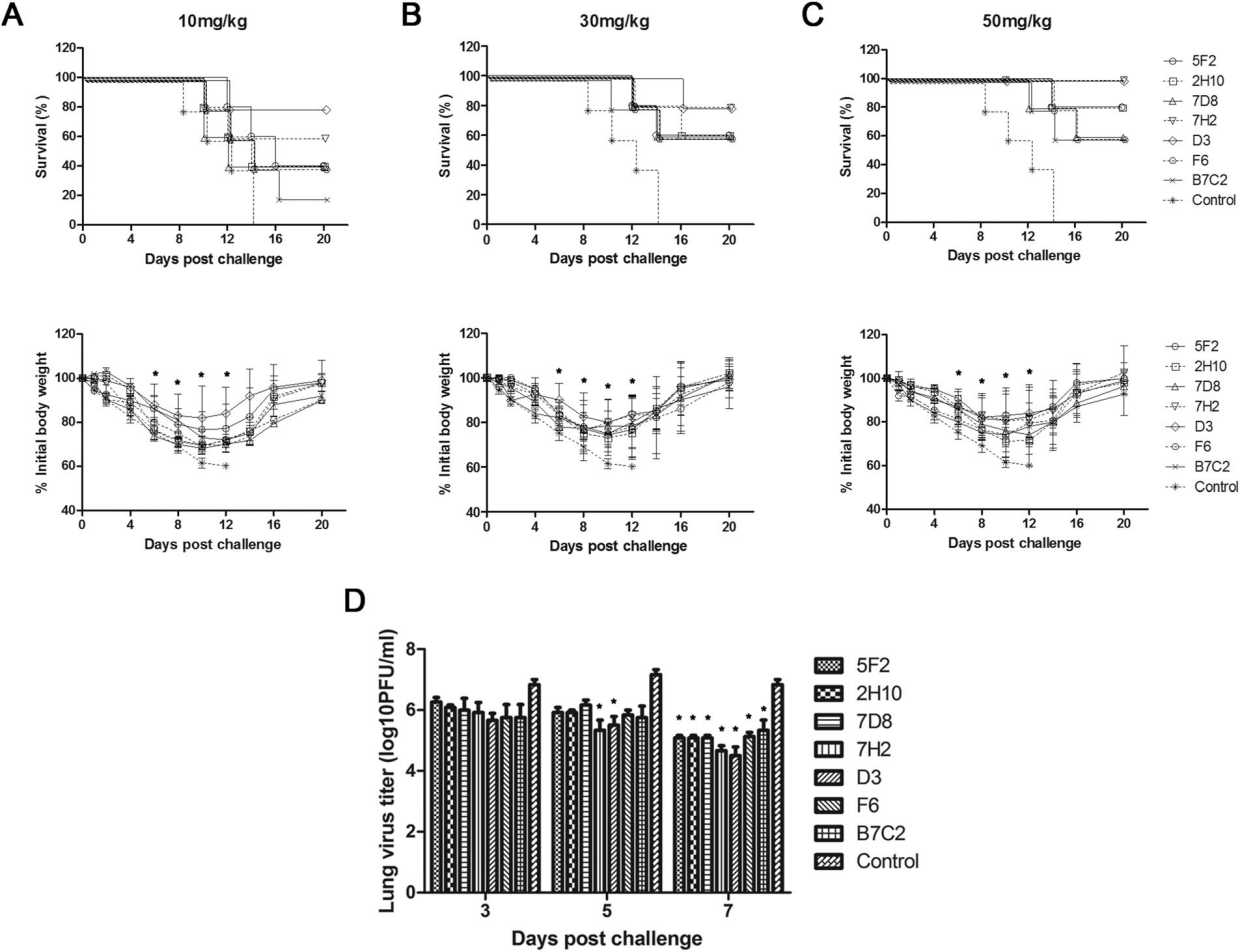
Therapeutic efficacy of N9 mAbs against a lethal H7N9 virus challenge in mice. BALB/c mice (*n* = 5 per group) were infected i.n. with 5 × LD_50_ of H7N9 SH/2/13 virus, followed by injection i.p. with indicated doses of each mAb 24 h later. An isotype-matched and irrelevant mAb was used as a negative control. (A-C) Survival (upper panel) and weight loss (lower panel) (*n* = 5 per group) were monitored for up to 20 days. (*, *p* < 0.05, 5F2 (10 mg/kg at 8, 10, 12d; 30 mg/kg at 10, 12d; 50 mg/kg at 6, 8, 10, 12d), 2H10 (30 mg/kg at 10, 12d; 50 mg/kg at 10, 12d), 7D8 (30 mg/kg at 10, 12d; 50 mg/kg at 6, 8, 10, 12d), 7H2 (30 mg/kg at 8, 10, 12d; 50 mg/kg at 6, 8, 10, 12d), D3 (10 mg/kg at 6, 8, 10, 12d; 30 mg/kg at 6, 8, 10, 12d; 50 mg/kg at 6, 8, 10, 12d), F6 (30 mg/kg at 10, 12d; 50 mg/kg at 10, 12d), B7C2 (30 mg/kg at 10, 12d; 50 mg/kg at 10, 12d) compared to the negative control by student *t*-test). (D) Lungs were collected on the 3, 5, and 7 d after infecting virus and viral titres were determined by titrating in MDCK cells. Titres were expressed as log10PFU/ml (*n* = 3); SD was shown with an error bar. Significant differences between titres measured in each group and control groups were shown as *, *p* < 0.05.

In the therapeutic study, survival (Figure 5, upper panel) and weight loss (Figure 5, lower panel) at different doses of mAbs were also monitored. As shown in Figure 5, the prophylactic effect of all mAbs was dose-dependent, with the greatest survival at the highest dose. 7H2 and D3 were the most effective mAbs with 100% protection at a dose of 50 mg/kg. Consistent with the *in vitro* observations, 7H2 and D3 had the best NI efficiency.

To determine whether mAbs 5F2, 2H10, 7D8, 7H2, D3, F6, and B7C2 reduced viral load in the lungs, mice were infected with H7N9 SH/2/13 virus, before or after treated with each mAb at 50 mg/kg, and sacrificed 3, 5, and 7 days after infection. Viral load in the lungs was measured by plaque assay (Figure 4D and 5D). High viral titres were detected in all groups of mice on day 3. Until on day 7 after infection, viral titres significantly decreased. 7H2 and D3 showed the best protection in both prophylaxis and therapeutic study, and also could reduce viral replication in the lungs.

Taken together, these results indicated that NA mAbs had prophylactic and therapeutic activity on lethal viral infections *in vivo*, and this correlated with *in vitro* functional properties of the mAbs.

## Discussion

The enzyme activity site of NA has long been the focus of antiviral drug designs. Now it also is used as a target for therapeutic antibodies and novel vaccines. The NA is present on the surface of virions and infected cells in the form of a tetramer. Since NA enzyme activity required native tetramer, this functional property is related to the immunogenicity [30,31]. Here we immunized mice with N9 NA DNA plasmid, as it may be difficult to produce NA with native structures because of the multiple inter-subunit interactions [30,32–34].

We screened seven strains of mAbs 5F2, 2H10, 7D8, 7H2, D3, F6, and B7C2 of H7N9 NA by the traditional hybridoma cell method, and located their epitopes on NA. We used the escape mutation method to screen the epitopes. The results showed that the seven mAbs bond to the lateral face of the NA “head” at different sites, and most were combined to the 250-loop and 370/400/430-loop antigen structure domains. The antigenic sites on NA are poorly understood compared with the influenza virus HA. For N9, a total of eight amino acid sites were determined [14]. Interestingly, four amino acids were also identified in our epitope mapping, indicating that these might be dominant regions. In addition, four NA amino acid sites identified by the D3 and 7H2 were first reported. These epitopes were highly conserved, target near 97.4% to 100% of H7 strains reported. The results suggested that the variation of these epitopes may be particularly important for the antigenic drift of the H7N9 virus, so it should be continuously monitored for human and animal hosts.

NA specific antibodies could reduce virus replication *in vitro*, mainly due to inhibition of enzyme activity of NA, which is usually the result of steric hindrance. When an antibody binds to the NA head, the antibody-NA complex prevents large substrates from entering the enzyme active pocket. If an antibody binds very closely to the enzyme activity pocket, small substrates could also be inhibited the entry of into the enzyme activity site. In some cases, since the natural substrate is large, antibodies binding some distance from the enzyme activity pocket also can inhibit the lysis of sialic acid. This inhibition of substrate cleavage prevents the elution of virion progeny from the host cell surface, resulting in restricting virus replication. Therefore, the antibodies that can inhibit NA activity may reduce the number of infected cells, which may explain that the NI titre is associated with the reduced infectivity in clinical studies [35]. The NI can be used as a key indicator to measure the ability of the NA antibody to inhibit the enzyme activity. We selected fetuin protein with a large molecular weight and NA-XTD with a small molecule weight as the substrates. Different mAbs exhibited different properties at the presence of the two substrates depending on their disparate binding sites. MAbs D3 and 7H2 binding to the 250-loop domain both effectively inhibited cleavage of both large and small substrates, and also reduced virus spread in plaque assays at a low concentration. MAbs 5F2 and 7D8 both binding to 370-loop with different inhibitory capacities in NI and plaque formation. 5F2 could effectively inhibit entry of two substrates into the enzyme activity pocket and prevent formation of plaques. However, 7D8 could only inhibit large substrate into an active site. MAb F6 binding around the 350-loop had some similar properties with 5F2. MAb B7C2 binding to 400-loop and 2H10 binding to 430-loop performed less effectively as well as other antibodies in the NI and plaque inhibition tests.

In the preventive trials, the intraperitoneal injection of 30 mg/kg or higher doses of mAbs 7H2 and D3 significantly protected mice from the lethal doses of H7N9 virus challenge. In the therapeutic trials, 7H2 and D3 were also the most effective protecting the mice from the lethal H7N9 virus for 24 h after challenge. As 7H2 and D3 could be capable of inhibition of NA enzymatic activity and of virus spread *in vitro*, they were likely to inhibit NA enzyme activity *in vivo*. In addition, we also compared mAbs 7H2 and D3 with some published N9 NA mAbs, such as 3c10-3 [11,12], 1E8, 2F6, 10F4, 11B2 [13]. Though the NI abilities of 7H2 and D3 were weaker than those of the published mAbs, 7H2 and D3 conferred 100% protection against H7N9 virus infection, and their binding activities were superior to the published antibodies. In the future, we would also determine the cross-protection of antibodies with heterologous influenza virus infection in mice.

So far, we have conducted long-term studies on the NA antigen of influenza viruses and confirmed that the NA antigen could not only induce a high level of specific antibody response in mice, but also confer protection against lethal infection with homologous or heterologous influenza viruses [15–19,21]. In addition, the combined immunization with the NA-DNA and HA-DNA could provide more effective protection than only NA-DNA or HA-DNA immunization, suggesting that the combination of NA and HA mAbs may be more effective than NA-mAb or HA-mAb alone [20]. Furthermore, antigenic drift, random genetic mutation of antigens, is best characterized in influenza viruses. The viruses undergo antigenic shift which may cause potentially severe disease and can spread quickly within a population of humans or animals. The antigenic variation of NA is much slower than that of HA, thus NA may be more important to provide effective protection against influenza virus infection [18].

In conclusion, we characterized seven mAbs against the H7N9 virus. Through the analysis of the antiviral properties *in vitro* and the ability of prevention and treatment *in vivo*, we found that D3 and 7H2 could well inhibit the N9 NA activity, plaque formation and protect mice against an H7N9 virus challenge. Results suggested that the NA mAbs could be used in the prevention and treatment of H7N9 influenza virus infection. The novel epitope we discovered here might play an important role in the development of influenza vaccines and anti-NA drugs.
